# Active methanogenesis during the melting of Marinoan snowball Earth

**DOI:** 10.1038/s41467-021-21114-6

**Published:** 2021-02-11

**Authors:** Zhouqiao Zhao, Bing Shen, Jian-Ming Zhu, Xianguo Lang, Guangliang Wu, Decan Tan, Haoxiang Pei, Tianzheng Huang, Meng Ning, Haoran Ma

**Affiliations:** 1 Key Laboratory of Orogenic Belts and Crustal Evolution, MOE, School of Earth and Space Science, Peking University, Beijing, China; 2 State Key Laboratory of Geological Processes and Mineral Resources, Institute of Earth Sciences, China University of Geosciences, Beijing, China; 3 State Key Laboratory of Oil and Gas Reservoir Geology and Exploitation & Institute of Sedimentary Geology, Chengdu University of Technology, Chengdu, China; 4 State Key Laboratory of Environmental Geochemistry, Institute of Geochemistry, Chinese Academy of Sciences, Guiyang, China; 5 University of Chinese Academy of Sciences, Beijing, China; 6 Institute of Mineral Resource, Chinese Academy of Geological Sciences, Beijing, China; 7 Present Address: Department of Atmospheric and Oceanic Sciences, School of Physics, Peking University, Beijing, China

**Keywords:** Palaeontology, Element cycles, Cryospheric science

## Abstract

Geological evidence indicates that the deglaciation of Marinoan snowball Earth ice age (~635 Myr ago) was associated with intense continental weathering, recovery of primary productivity, transient marine euxinia, and potentially extensive CH_4_ emission. It is proposed that the deglacial CH_4_ emissions may have provided positive feedbacks for ice melting and global warming. However, the origin of CH_4_ remains unclear. Here we report Ni isotopes (δ^60^Ni) and Yttrium-rare earth element (YREE) compositions of syndepositional pyrites from the upper most Nantuo Formation (equivalent deposits of the Marinoan glaciation), South China. The Nantuo pyrite displays anti-correlations between Ni concentration and δ^60^Ni, and between Ni concentration and Sm/Yb ratio, suggesting mixing between Ni in seawater and Ni from methanogens. Our study indicates active methanogenesis during the termination of Marinoan snowball Earth. This suggests that methanogenesis was fueled by methyl sulfides produced in sulfidic seawater during the deglacial recovery of marine primary productivity.

## Abstract (web summary)

The deglaciation of Marinoan snowball Earth (~635 Myr ago) has been associated with potentially extensive CH_4_ emissions in relation to transient marine euxinia. Here, the authors find that active methanogenesis occurred during the termination of Marinoan snowball Earth, fueled by methyl sulfide production in sulfidic seawater.

## Introduction

The Marinoan snowball Earth glaciation (~650–635 Myr ago) represents one of the most severe ice ages in Earth’s history, during which ice sheets may have reached tropical latitudes and the Earth may have remained frozen for millions of years^1^. Geological, geochemical, and paleontological data indicate that the termination of the Marinoan glaciation was immediately followed by atmosphere and ocean oxygenation, as well as the diversification of eukaryotes in the earliest Ediacaran Period^2–4^. The coincidence of these major events suggests that there was possible causal linkage among global glaciation, environmental change, and biological evolution. To understand the consequences of the Marinoan snowball Earth, it is essential to reconstruct the sequence of events that occurred during deglaciation.

The deglaciation of Marinoan snowball Earth was associated with intense continental weathering, recovery of marine productivity, development of oceanic euxinia, and global precipitation of cap carbonate^5–7^. The snowball Earth hypothesis suggests that high atmospheric CO_2_ level was accumulated during the snowball Earth. The CO_2_ accumulation generated a super-greenhouse condition that resulted in the rapid meltdown of ice on the Earth’s surface^8^. Such a high atmospheric CO_2_ level would trigger intense continental weathering, resulting in the global precipitation of cap carbonate^9^. Substantial continental weathering is supported by a positive excursion in Mg isotopes (e.g., δ^26^Mg anomaly found in the siltstone between glacial debris and the cap carbonate^5^). Nitrogen isotopes suggest active biogeochemical cycle during the glacial episodes^10^. Because chemical weathering could transport abundant nutrients and a surge of sulfate into the ocean, it is inferred that the deglaciation includes an increase in organic matter production and microbial sulfate reduction (MSR) in seawater^7^. High primary productivity and marine euxinia are evidenced by the abundant deposition of syndepositional pyrite in the topmost of Nantuo Formation throughout the Yangtze Block, South China. These pyrites show relatively high sulfur isotope values (δ^34^S_py_) and a nonzero multiple sulfur isotope compositions (Δ^33^S). Sulfur isotopic modeling suggests that high δ^34^S_py_ values and high pyrite content result from active MSR in seawater, generating an episode of oceanic euxinia prior to cap carbonate precipitation^7^.

Another event that might occur during deglaciation is massive CH_4_ emission. It has been proposed that destabilization of CH_4_ clathrates may have provided positive feedbacks for the termination of Marinoan snowball Earth ice age and dramatic climate changes^11,12^. Geological evidence of deglacial CH_4_ emission includes sporadic occurrences of extremely low (<−40‰) carbonate carbon isotopes (δ^13^C_carb_) in some cap carbonate^13^. while some cap carbonate fabrics, such as tepee-like structures, resemble structures in cold CH_4_ seeps^11,13^.

However, it remains unclear what the source of CH_4_ may have been, and how CH_4_ emission is linked with marine biogeochemical cycles during deglaciation. CH_4_ clathrate formation requires an ample supply of organic matter. Here, we suggest that the deglacial recovery of primary productivity would favor methanogenesis and CH_4_ accumulation in clathrate. In this study, we tested this hypothesis by using nickel (Ni) isotopes and yttrium-rare earth elements (YREE) compositions. We analyzed Ni isotopes and YREE compositions of syndepositional pyrite in the deglacial deposits from the Nantuo Formation, South China. The geochemical data indicate active methanogenesis during the melting of Marinoan snowball Earth and provides important perspectives on the termination of Marinoan snowball Earth ice age.

## Results

### Geological setting and the Nantuo pyrite concretions

**Fig. 1:**
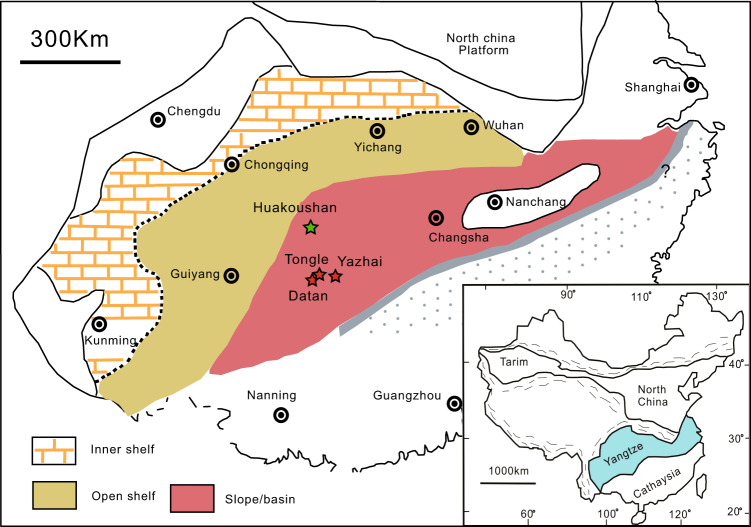
The paleogeographic map of South China Block in late Neoproterozoic Era^7^. The green star marks the location of Huakoushan section (slope environment). The red stars mark the locations of Yazhai, Tongle, and Datan sections (basin environment).

**Fig. 2:**
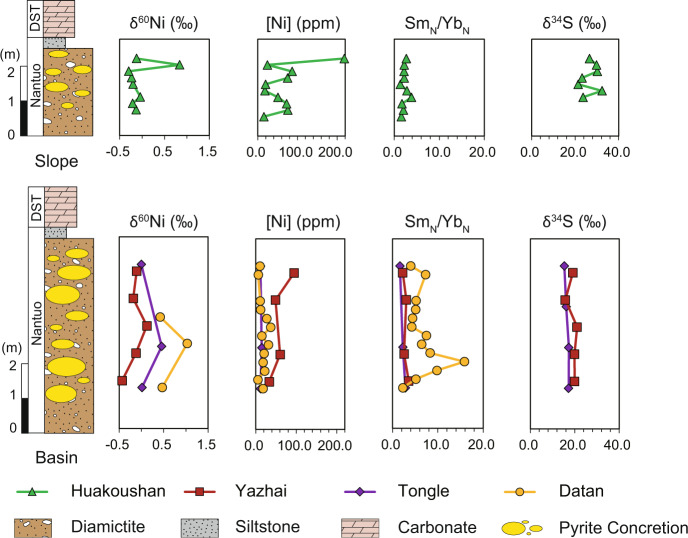
The stratigraphic profiles of geochemical data. Stratigraphic variations of δ^60^Ni, Ni concentration, Sm/Yb (normalized by post-Archean Australian shale, PAAS), and δ^34^S of pyrite concretions in the upper Nantuo Formation. The δ^34^S values are from Lang et. al.^7^. DST here refers to Doushantuo Formation.

The Cryogenian deposits in the Yangtze Block are composed of, in ascending order, the Chang’an, Fulu, Datangpo, and Nantuo Formations. The Nantuo Formation (ca. 650–635 Ma) is correlated with the glacial deposition of the Marinoan global glaciation^14,15^. The Nantuo Formation is conformably overlain by a 3–6 m thick cap carbonate in the basal Doushantuo Formation. In the Yangtze Block, the Nantuo Formation attenuates from 2000 m of basin facies in the southeast (present orientation) to a few meters of shelf facies in the northwest (Fig. 1). Non-glacial marine and distal glaciomarine facies associations are identified in the upper part of the Nantuo Formation throughout the Yangtze Block, suggesting that the deglaciation had occurred before the cap carbonate precipitation^16^. Abundant pyrite concretions are discovered in the top 0.5–10 m of the Nantuo Formation (Fig. 2)^7^. Their abundance and size decrease from the basin to shelf sections^7^.

In this study, pyrite concretions from four sections were analyzed, including one slope (Huakoushan) and three basin sections (Tongle, Yazhai, and Datan; Fig. 1). Pyrite concretions occur as elliptical nodules with their long axis parallel to the bedding surface (Fig. S2 in SI). The diameter of pyrite concretions can be as large as 20 cm in basin sections, but is reduced to ~1 cm in shelf regions. Pyrite nodules are composed of densely packed euhedral pyrite crystals, no framboidal pyrite, or framboidal cores are identified^7^.

### Ni isotopes and YREE data

**Fig. 3:**
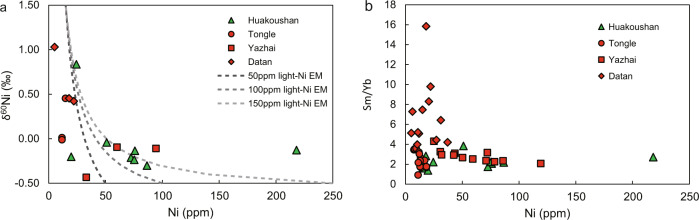
The relationships between Ni concentration, Ni isotopes, and rare earth element pattern of the Nantuo pyrite samples. **a** Cross-plot of Ni concentration versus δ^60^Ni. There is an anticorrelation between Ni concentrations and δ^60^Ni values. Dash lines represent the relationship between Ni concentration and isotopes in a theoretical mixing model. Here, δ^60^Ni values of the two end-members are −0.5‰ and +1.5‰, respectively. The Ni concentration of the high-δ^60^Ni end-member is fixed to 20 p.p.m., while that of low-δ^60^Ni end-member is assigned to 50, 100, and 250 p.p.m., respectively. The anticorrelation between Ni concentration and δ^60^Ni could be explained by a binary mixing model. **b** Cross-plot of Ni concentration versus Sm_N_/Yb_N_. Here, Sm and Yb are used to represent middle rare earth element (MREE) and heavy REE (HREE) respectively. An anticorrelation is shown between Ni concentration and MREE/HREE. REE data are normalized to post-Archean Australian shale (PAAS). Green triangles, red circles, red squares, and red diamonds represent the Huakoushan, Tongle, Yazhai, and Datan sections, respectively.

**Fig. 4:**
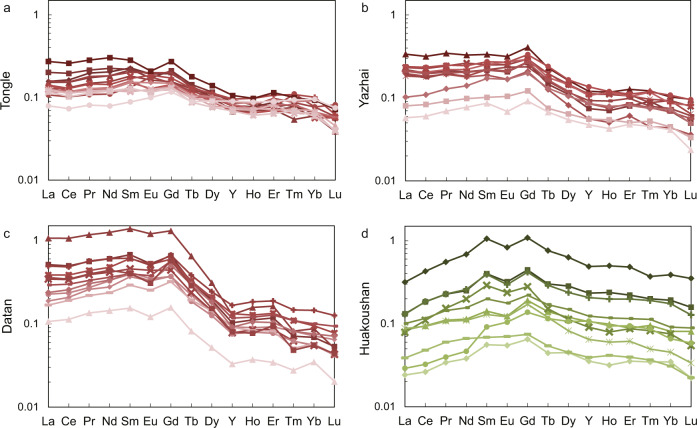
Rare earth element pattern of the Nantuo pyrite. Rare earth element (REE) data are normalized to post-Archean Australian shale (PAAS). Samples formed in the basin environment (Tongle, Yazhai, and Datan section) are strongly depleted in HREE. Samples from the slope region (Huakoushan section) enrich in MREE.

δ^60^Ni values of the Nantuo pyrites range from −0.5 to +1.0‰ (Fig. 2 and Table S1 in SI). There is an anticorrelation between δ^60^Ni and Ni concentration, i.e., samples with high δ^60^Ni values are characterized by low Ni concentrations (Fig. 3a). The YREE pattern for pyrite (normalized to post-Archean Australian shale, PAAS) is facies dependent (Fig. 4 and Table S2 in SI). Pyrites from the basin sections are strongly depleted in heavy REE (HREE), showing light REE (LREE) ≈ MREE > HREE pattern, while those from the slope section enrich in middle REE (MREE). All pyrite samples show a slight Eu negative anomaly, but no yttrium anomaly. There is an inverse correlation between Ni concentration and MREE/HREE (Fig. 3b), but Ni concentration and MREE/LREE do not show any correlation (Fig. S1).

## Discussion

Pyrite could be formed by the following processes: direct precipitation from seawater, post-depositional precipitation in hydrothermal activity, or in situ (authigenic) precipitation in porewater during early diagenesis. Direct precipitation from seawater could be ruled out since the Nantuo pyrite consists of euhedral crystals, and no framboidal pyrite or framboidal core within pyrite were found (Fig. S3 in SI)^7^. The hydrothermal origin is also unlikely since the REE patterns of Nantuo pyrites contrast with that of hydrothermal pyrite, which is commonly characterized by a positive Eu anomaly with a flat REE distribution pattern^17–19^.

Petrological evidence indicates that the Nantuo pyrite concretions were precipitated within sediment porewater^7^. The euhedral pyrite crystal in glacial diamictite suggests an authigenic origin of the pyrite (Fig. S3 in SI)^7^. Meanwhile, the tightly packed pyrite crystals are either cemented by silica or wrapped by siliciclastic matrix (Fig. S3 in SI), suggesting the pyrite formation predating sediment compaction. Thus, pyrite samples analyzed in this study are mainly formed during early diagenesis, recording the geochemical signal of porewater.

The authigenic pyrite formation in porewater requires Fe supply in sediment. Theoretically iron oxides are thermodynamically unstable and pyrite precipitation is spontaneous in sulfidic water. However, no framboidal structure or framboidal core is found in Nantuo pyrite, which is in contrast with the direct precipitation from sulfidic seawater. Here, we suggest that reactive Fe is supplied by microbial iron reduction (MIR) of ferrihydrites in sediment. Recent studies indicate active MIR in porewater with the water column remaining sulfidic^20,21^, confirming transportation of particulate iron oxides through sulfidic water column. The absence of depositional pyrite formation in sulfidic water column may result from kinetic prohibition of pyritization of particulate iron oxides^21^.

In addition, high δ^34^S values up to +40‰ of the Nantuo pyrite (Fig. 2) indicate that MSR mainly occurred in the water column with seawater pervasively sulfidic (H_2_S-enriched), and the Nantuo pyrites were mainly formed in sediment porewater^7^. High δ^34^S values could be explained by either MSR in closed porewater (i.e., the Rayleigh distillation process) or MSR in bottom seawater with H_2_S diffused into porewater. However, the former scenario could only contributes to pyrite content of <0.1 wt.%, which cannot explain abundant pyrite precipitation up to 10 wt.% (ref. ^7^). As a result, MSR may have occurred in the bottom seawater, while the pyrite was formed during early diagenesis with H_2_S diffusion from sulfidic seawater.

**Fig. 5:**
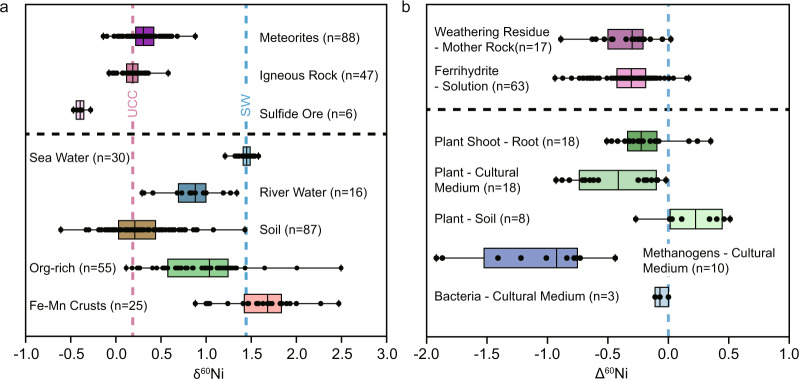
Compilations of the Ni isotopes data. **a** Boxplots showing the Ni isotopic compositions of different reservoirs^29–31,42,43,67–70^. The box showing the range for δ^60^Ni value between upper and lower quartiles. The vertical line inside the box represents the median value, while that outside the box represents the maximum and minimum value. The red and blue dash lines represent the upper continental crust (UCC) and modern seawater compositions, respectively^28,66^. **b** Boxplot showing the Ni isotopic fractionations in various geochemical processes^29,38^. The weathering process preferentially dissolves heavy Ni, and laboratory experiment shows preferential absorption of light Ni in ferrihydrite precipitation^41^. Plants and methanogens preferentially absorb light Ni (ref. ^25^).

In this study, we focus on Ni isotopes. Ni is the core metal in the porphyrin ring of the coenzyme for methanogenesis^22,23^. Methanogens preferentially utilize isotopically light Ni with an isotopic fractionation up to −0.8‰ (ref. ^24^). Other microbes also utilize Ni, but there is no evidence showing that other microbes could fractionate Ni isotopes (Fig. 5b)^24^. Terrestrial plants can fractionate Ni isotopes^25^, but it is reasonable to argue that Ni isotope fractionation in Proterozoic samples cannot be explained by the biological fractionation of land plants^26^. Thus, we infer that Ni isotopes can trace methanogenesis in Proterozoic.

It should be noted that interpretation of Ni isotopes might be complicated by other abiotic processes. Igneous rocks have nearly homogeneous Ni isotopic compositions with δ^60^Ni ≈ +0.15‰ (Fig. 5a)^27–31^, while the average δ^60^Ni value for meteorites is around +0.23‰ (refs. ^32–34^). Continental weathering fractionates Ni isotopes with heavy Ni being preferentially released into solution. As such, modern seawater has δ^60^Ni of ~+1.4‰ (refs. ^28,35^). The δ^60^Ni values for euxinic Black Sea deep water could be up to ~+2.0‰ (ref. ^36^), perhaps caused by the Mn recycling near the redoxcline. In addition, Fe-oxyhydroxide and sulfide precipitation preferentially absorb light Ni isotopes^29,37–43^, which would interfere with Ni isotopic fractionation by methanogenesis. These biological and abiotic processes can be further evaluated by the YREE system.

**Fig. 6:**
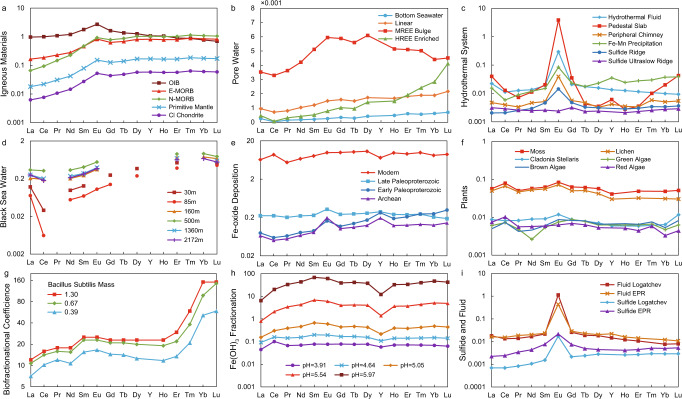
Compilations of rare earth element pattern. All rare earth element (REE) data are normalized to post-Archean Australian shale (PAAS)^44^. **a** REE pattern of igneous rocks. All samples show Eu positive anomalies. All except for oceanic island basalt (OIB) show light REE (LREE)-depleted pattern; **b** REE pattern of porewater. REE pattern of the bottom water is similar to that of seawater. The middle REE (MREE) bulge type is found in Fe rich cores. The heavy REE (HREE)-enriched type is found in Fe lean cores. All samples slightly enrich in HREE^45^; **c** REE pattern of hydrothermal fluids and precipitates. Most samples show a flat REE pattern, except for the pedestal slab near chimney showing a MREE-depleted pattern^17,19,50^. All samples show a positive Eu anomaly except for sulfide from ultraslow spreading ridge. Pedestal slab and Fe–Mn precipitation show a slight Y enrichment^50^. **d** REE pattern for Black Seawater. Strong Ce negative anomaly could be characterized in shallow water. Deep water shows flat REE pattern with slight HREE enrichment^46^. **e** REE pattern of Fe-oxide deposition. Modern samples are ferrihydrite precipitation on seafloor, while samples from Paleoproterozoic and Archean era are banded-iron-formation (BIF). Modern samples show flat pattern with slight Ce positive anomaly and negative Y anomaly. BIF samples show positive Eu and Y anomaly^48^. **f** REE pattern for modern primordial plants including lichen, algae, and moss.^53,54^ All samples show a flat REE pattern, except for green algae that is slightly depleted Nd (ref. ^54^). **g** Fractional coefficient for microbes. Different lines show fractional coefficient for different biomass concentration. Microbes preferentially absorb HREE^57^. **h** REE fractionation during Fe(OH)_3_ precipitation. All data show LREE-depleted pattern with a slight Y negative anomaly. Smaller fractionation is observed in acidic environment^47^. **i** Comparison of REE pattern for fluid end-member and sulfide from Logatchev (mid-ocean ridge in Atlantic Ocean) and east Pacific rise (EPR)^19^. The REE pattern for sulfide and fluid end-member is generally similar. A smaller positive Eu anomaly and slight HREE enrichment might be caused by contamination of seawater^19^.

The REE pattern (normalized to shale) of igneous rocks show a LREE-depleted pattern (LREE < MREE ≈ HREE; Fig. 6a)^44^, while the modern surface seawater is characterized by the HREE-enriched distribution and a negative Ce anomaly (Fig. 6b)^45^. By contrast, REE of anoxic porewater displays either a flat (i.e., LREE ≈ MREE ≈ HREE), HREE-enriched or MREE-enriched pattern (Fig. 6b)^45^. In the Black Sea, strong Ce negative anomaly can be seen in shallow water (<100 m), due to the active Mn redox cycle, but a relatively flat REE pattern can be seen in deep water (Fig. 6d)^46^. Precipitation of Fe-oxyhydroxides causes a negative yttrium anomaly and displays a flat or LREE < MREE ≈ HREE pattern (Fig. 6h)^47^. Ancient Fe-oxide deposits (i.e., BIF) show positive Eu and Y anomalies, while modern ferrihydrite deposition shows a positive Ce anomaly and a negative Y anomaly (Fig. 6e)^48,49^. The REE patterns for hydrothermal fluid and hydrothermal sulfides are flat with a characteristic Eu positive anomaly, but the amplitude of Eu anomaly for sulfide is smaller than that of hydrothermal fluid (Fig. 6c)^17–19^. Hydrothermal fluids and hydrothermal precipitates in the mid-ocean ridges are strongly enriched in Eu (Fig. 6c)^17,19^. Pedestal slab near the chimney shows a MREE-depleted pattern and is enriched in both Eu and Y (Fig. 6c)^50^. The similar REE pattern between hydrothermal fluid and hydrothermal pyrite implies that pyrite precipitation did not fractionate REE (Fig. 6i)^18,19,51,52^. A weak Eu enrichment and a slight HREE-enriched pattern in sulfide may be explained by mixing with low temperature seawater^19^. It is also reasonable to speculate that Yttrium would not be fractionated by sulfide precipitation since no Yttrium anomaly is found in peripheral chimney (Fig. 6c)^50^. Primordial plants, including moss, lichen, and algae, show a flat YREE pattern, except for green algae which depletes in Nd (Fig. 6f)^53,54^. Since microbes preferentially absorb HREE, organic matter produced by microbes (i.e., biomass of microbes) is characterized by LREE ≈ MREE < HREE (Fig. 6g)^55–57^. Therefore, although both methanogenesis and Fe-oxyhydroxides precipitation preferentially utilize/scavenge light Ni isotopes, methanogenesis can be distinguished by the HREE enrichment and the absence of Y anomaly.

The combination of Ni isotopes and YREE data can be used to trace methanogenesis during the Nantuo pyrite precipitation. Contamination of clay minerals could alter both Ni and REE concentrations. Only samples from the slope region (HKS section) show positive correlations between Ni concentration, MREE/HREE and Al, Ti contents (Fig. S4 in SI), suggesting the potential contamination from clays. For the same reason, absence of such correlations can rule out clay contamination in all basin samples (Fig. S5 in SI). Thus, both Ni and YREE in the basin samples appear to be derived from pyrite.

δ^60^Ni values of the Nantuo pyrites show a wide range of variation. There is a negative correlation between δ^60^Ni and Ni concentration (Fig. 3a). A similar negative correlation is observed between MREE/HREE ratio and Ni concentration (Fig. 3b). These anticorrelations could be explained in two ways: (1) the Rayleigh fractionation in a closed system during pyrite precipitation, and (2) a binary mixing between two end-members with different δ^60^Ni values and Ni concentrations.

In the first scenario, i.e., the Rayleigh process in a closed system, both light Ni isotopes and HREE should be preferentially removed from porewater, elevating δ^60^Ni value and depleting HREE in porewater. We suggest that pyrite precipitation cannot be the major process for removing porewater Ni in the Rayleigh distillation. Although light Ni would be preferentially removed, pyrite precipitation does not fractionate REE^18,19,51,52^. Alternatively, methanogens preferentially utilize both light Ni and HREE (Figs. 3b and 6g)^24,55–57^, lowering the Ni concentration, and elevating δ^60^Ni and Sm/Yb ratio of porewater. Therefore, pyrite precipitation in porewater with active methanogenesis would generate anticorrelations of Ni concentration, with both δ^60^Ni and Sm/Yb ratio. However, we suggest that the Rayleigh distillation in a close porewater system is unlikely, because high pyrite content requires sufficient connection between porewater and seawater, allowing sustained diffusion of seawater H_2_S (ref. ^7^).

Alternatively, the anticorrelation could also be explained by the binary mixing of two sources (see text in SI)^58,59^. One end-member has a lower δ^60^Ni value and is enriched in HREE (LREE ≈ MREE < HREE), while the other is characterized by a higher δ^60^Ni value and a HREE-depleted pattern (LREE ≈ MREE > HREE). Igneous rock fragment has a low δ^60^Ni value, but shows a LREE < MREE ≈ HREE pattern (Fig. 6a)^44^, and thus cannot be the low-δ^60^Ni end-member. The interference of Mn cycle can be ruled out because of the absence of Ce anomaly^46^. Primordial plants could not be the end-member either, since they show a flat REE pattern (Fig. 6f)^53,54^. Contamination of Fe-oxyhydroxides could also be excluded by the absence of yttrium anomaly (Fig. 4). Another candidate of the low-δ^60^Ni end-member is the biomass of methanogens, i.e., organic matter produced by methanogens^24^. Because microbes preferentially absorb HREE and methanogens prefer light Ni isotope^55–57^, the biomass of methanogens would have a LREE ≈ MREE < HREE pattern and a low δ^60^Ni value (Fig. 6g).

The heavy δ^60^Ni end-member with low Ni content might be represented by porewater or seawater that has been modified by methanogenesis. Wherever methanogenesis occurs, absorption of HREE and preferential utilization of light Ni by methanogens would result in a depletion of HREE and an enrichment of heavy Ni (refs. ^55,56^). During syndepositional pyrite precipitation in sediment porewater, degradation of methanogens would provide both light Ni and HREE. Various degree of biomass degradation during pyrite precipitation would generate a negative correlation between δ^60^Ni and Ni concentration, and between MREE/HREE and Ni concentration (Fig. 3).

Ni isotopes and YREE data of the Nantuo pyrites indicate degradation of organic matter that was derived from methanogenesis, and the release of light Ni and HREE, suggesting active methanogenesis during pyrite precipitation. Methanogenesis could occur in both porewater and seawater. However, syndepositional origin of the Nantuo pyrite requires active MIR in sediment porewater, which would outcompete methanogenesis^7^. In addition, the limited supply in methyl sulfide in the MIR zone would lower the efficiency of methanogenesis in sediment porewater. Thus, methanogenesis may not be a predominant microbial process in sediments.

**Fig. 7:**
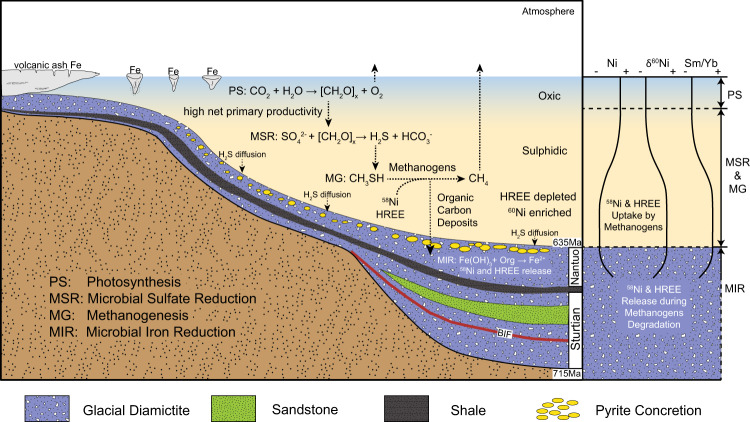
Schematic model showing the biogeochemical cycles during the termination of Marinoan snowball Earth in the Yangtze Block. The recovery of primary productivity provided abundant organic matter in the euphotic zone by photosynthesis (PS), fueling microbial sulfate reduction (MSR) in the water column. Seawater MSR sustained sulfidic condition.^7^ Active methanogenesis (MG) might be fueled by methyl sulfide that was produced by H_2_S-methylation in sulfidic seawater. Since methanogens preferentially absorb light Ni and heavy rare earth element (HREE), seawater was characterized by higher δ^60^Ni and displayed a HREE-depleted pattern. Sinking of particulate biomass of methanogens into sediments sustained microbial iron reduction (MIR), which converted ferric Fe (Fe^3+^) to Fe^2+^, and released absorbed light Ni and HREE in porewater. Syndepositional pyrite precipitation in sediment porewater would incorporate both seawater and porewater signals, and various degree of degradation of methanogen-derived organic matter led to a negative correlation between Ni concentration and δ^60^Ni.

Instead, we suggest that active methanogenesis might occur in sulfidic seawater. Theoretically, methanogenesis would be inhibited in the presence of sulfide^60^. However, the methanogenesis and MSR zones could converge with the presence of methyl sulfides, which can serve as a noncompetitive substrate for methanogens. Methyl sulfides, such as dimethyl sulfide (CH_3_SCH_3_) and methanethiol (CH_3_SH), could be abundantly generated in sulfidic water via sulfide methylation^61^. It is highly plausible that active methanogenesis together with MSR occurred in the sulfidic water column during the deglacial recovery of primary productivity (Fig. 7).

In the deglacial sulfidic seawater with presumably ample supply of methyl sulfide via sulfide methylation, methanogens preferentially utilize light Ni isotopes and scavenge HREE, driving seawater enriched in heavy Ni isotopes and depleted of HREE. If methanogenesis-derived organic matter enriched in light Ni, and HREE was delivered to the sediment and fueled MIR in sediment porewater, various degree of degradation would generate negative correlations between δ^60^Ni and Ni content, and between Sm/Yb and Ni content (Fig. 3).

Active methanogenesis in sulfidic seawater also implies an unusual microbial metabolic loop during the deglaciation of Marinoan snowball Earth. In this scenario, high organic matter production would be induced by intense continental weathering, while decomposition of primarily produced organic matter depleted O_2_ in seawater and allowed active MSR in the water column, sustaining the sulfidic conditions^7^. Methyl sulfide formed in sulfidic seawater may function as a noncompetitive substrate for methanogens^61–64^. Active methanogenesis might also contribute to the organic matter production within sulfidic water column and sustain MIR in sediment porewater by providing additional organic substrate.

As such, during the deglacial recovery of primary productivity, photosynthesis took place in the euphotic zone, MSR and methanogenesis occurred in sulfidic water column, and MIR dominated in sediment porewater (Fig. 7). Such redox zonation is different from the canonical model with the MSR zone underlying the MIR zone, but overlying the methanogenesis zone in sediments^60^, and implies a critically different redox profile. More importantly, such microbial metabolic network might efficiently remineralize organic matter, lowering the δ^13^C values of seawater before cap carbonate precipitation.

Finally, it is also inferred from this study that active CH_4_ production occurred before cap carbonate precipitation. On one hand, most CH_4_ produced in seawater might be released into atmosphere, exacerbating the greenhouse condition during deglaciation. On the other hand, CH_4_ production near the seafloor or within sediments was likely to be stored as CH_4_ clathrate^65^. The following clathrate destabilization and CH_4_ oxidation would provide excessive ^12^C at local or regional scale, leaving traces of methane-derived carbon isotope signal in cap carbonate^13^.

## Methods

### Sample preparation and digestion

Fresh outcrop samples were used for chemical composition analysis. In order to minimize the influence of weathering and silicate component, only samples with euhedral pyrite crystals were used. Pyrite aggregates were selected under stereoscope. Pyrite nodules were directly knocked down from the host rock. Selected pyrites were digested using the mixture of concentrated HNO_3_ and HCl, until the liquid was clear and only a little insoluble silicate clast left. The liquid was evaporated to dry, then completely dissolved in the mixture of 1 mol/L HCl and 1 mol/L HNO_3_. After centrifugated, the supernatant was used for further analysis. Samples digestion was conducted at the Isotope Geochemistry Laboratory of China University of Geosciences, Beijing.

### Major and trace elements analyses

Concentrations of major and trace elements were measured by ICP-OES (Spectro Blue Sop) at Peking University. REE concentrations were measured by inductively coupled plasma mass spectrometer (ICP-MS; Perkin Elmer NexION300) in Chinese Academy of Geological Sciences. Element concentration for the pyrite was calculated based on whole rock mass. The reproducibility for major and minor elements (including Fe, Ni, and REE) is better than 5%.

### Nickel isotope analysis

Samples containing ~200 ng Ni was used for Ni purification and isotope analysis. Before Ni purification, ^61^Ni–^62^Ni double spike was mixed with the sample solution. The optimized ratio of ^62^Ni_spike_:^58^Ni_sample_ is ~1.3. Five purification procedures were used to suppress the interference from Fe, Zn, Mg, Ca, and other elements (see SI)^66^. The yield of Ni for the total procedure is >90%. The procedure blank for ^60^Ni is negligible relative to sample signal (1–2 V). Four geological standard materials (BHVO-2, BCR-2, NOD-A-1, and NOD-P-1) were processed with sample for chemical purification. The purified Ni solution with Fe/Ni, Zn/Ni, Ca/Ni, and Mg/Ni <0.01 was analyzed on a Nu plasma II High Resolution Multi-Collector ICP-MS at State Key Laboratory of Environmental Geochemistry in Institute of Geochemistry, Chinese Academy of Sciences, Guiyang. Faraday cups L5, L4, L2, AX, H2, H4, H6, H7, and H8 are used to collect ^57^Fe, ^58^(Fe, Ni), ^59^Co, ^60^Ni, ^61^Ni, ^62^Ni, ^63^Cu, ^64^(Ni, Zn), ^65^Cu, and ^66^Zn, respectively. The isobaric interference from ^58^Fe and ^64^Zn were corrected by ^57^Fe and ^66^Zn, respectively. ^58^Ni, ^60^Ni, ^61^Ni, and ^62^Ni are used for analysis here. Ni isotopic values are reported by δ-notation as ‰ deviation relative to NIST 986 standard: δ^x^Ni = [(^*x*^Ni/^58^Ni)_sample_/(^*x*^Ni/^58^Ni)_NIST SRM 986_ − 1] × 1000, where *x* refers to 60, 61, or 62. Each measurement contains three blocks, each block contains ten cycles. The integration time for each cycle is 10 s. The external precision of long-term measurement is 0.09‰ based on the multiple determination of reference materials. All measured values for the geological referencce materials are consistent with the published value (Fig. S6 in SI).

## Supplementary information

Supplementary Information

## Data Availability

All data in this study are available from the corresponding author via email upon reasonable request.
