# Supplementary material for: Active methanogenesis during the melting of Marinoan snowball Earth
Source: Nat Commun. 2021 Feb 11;12:955. doi: 10.1038/s41467-021-21114-6 (PMC7878791; doi:10.1038/s41467-021-21114-6)
Supplement: Supplementary file 1 — Supplementary Information [file 41467_2021_21114_MOESM1_ESM.pdf]

Supplementary Information

For the paper "Active Methanogenesis during the Melting of Marinoan Snowball Earth"

by Zhouqiao Zhao *et al*.

Contents:

Supplementary Notes 1. Geological Background and Sample Description

Supplementary Notes 2. Calculation for Mixing Model

Supplementary Notes 3. Calculation for Rayleigh Distillation

Supplementary Notes 4. Method for Nickel Purification

Supplementary Table S1. Nickel isotope data

Supplementary Table S2. REE component and element concentration data (in ppm)

Supplementary Table S3. Ni purification procedure

Supplementary Figure S1: Cross-plot of Ni concentration versus Sm_N/La_N for Nantuo pyrite samples.

Supplementary Figure S2: Outcrop photographs for pyrite concretions.

Supplementary Figure S3: Micrographs for Nantuo pyrite.

Supplementary Figure S4: Cross-plot of Ni concentration, Sm_N/Yb_N and MREE* versus Al_2O_3 and Ti contents for slope samples.

Supplementary Figure S5: Cross-plot of Ni concentration, Sm_N/Yb_N and MREE* versus Al_2O_3 and Ti contents for basin samples.

Supplementary Figure S6: δ^{60}Ni value for geological reference material

Supplementary Reference

Supplementary Notes 1. Geological Background and Sample Description

The South China Block (SCB) consists of the Yangtze Block in the modern northwest and Cathaysia Block in the modern southeast[1, 2]. The amalgamation of the two blocks occurred at ~830–820 Ma, followed by a rifting and thermos-subsidence cycle in late Neoproterozoic.

The early rifting stage is recorded by the Liantuo/Chengjiang formations in the shallow water facies and by the Banxi/Danzhou/Xiajiang groups in the deep water facies. The waning rifting of the SCB is represented by the Cryogenian successions which range from 10s meters thick in the shelf to 1000s meters thick in the basin. The thermo-subsidence succession is characterized by widespread passive margin carbonate deposits, including Ediacaran Doushantuo and Dengying/Liuchapo/Laobao formations.

The Cryogenian succession in SCB can be divided into three units, with two glacial successions and an interglacial interval between them[3, 4]. The first glacial succession is represented by Jiangkou glacial deposits that consist of, in ascending order, Chang'an Formation and the lower part of the Fulu Formation in basin environment and Gucheng/Tiesiao/Dongshanfeng (GTD) Formation in slope region. The Jiangkou glacial deposits were radiometrically dated between 715.9 ± 2.8 Ma and 658.8 ± 0.5 Ma, and thus the Jiangkou glaciation is correlated with the Sturtian snowball Earth glaciation.

The interglacial interval is represented by the topmost of Fulu Formation in basin and Datangpo Formation in the slope. The Datangpo Formation is constituted by muddy sandstone/siltstone, shale or silty mudstone, whereas the topmost of the Fulu Formation is mainly composed of laminated siltstones.

The upper glacial deposits are represented by the Nantuo Formation[5]. In the Yangtze Block, the Nantuo Formation shortens from 2000 meters of basin facies in the southeast (present orientation) to a few meters in thickness of shelf facies in the northwest. An U-Pb age of 635 ± 0.6Ma was reported from the top of Nantuo Formation, thus the Nantuo Formation corresponds with the Marinoan glacial deposits[6, 7]. The Nantuo Formation unconformably overlies Liantuo/Chengjiang formations in shallow facies, while it conformably overlies the interglacial deposits (the Datangpo Formation or the top of the Fulu Formation) in deep water facies. The Nantuo Formation is conformably overlain by a 3-6 meter thick cap carbonate in the basal Doushantuo Formation.

Two glacial episodes were recognized in the Nantuo Formation: The first glacial interval is

recorded by massive coarse-grained diamictite in the lower part of Nantuo Formation, while the second glacial episode is represented by the re-appearance of diamictite in the middle parts of the Nantuo Formation[4]. Approximately 10-meter-thick siltstone/shale were deposited between the two units of glacial deposits, suggesting an non-glacial period within the Marinoan glaciation. In the upper most of the Nantuo Formation, ~10s meter-thick pebbly sandstone/siltstone units represents the deglacial period of the Marinoan glaciation which was supported by isotopically heavy Mg values ($\delta^{26}Mg \approx 1.0‰$), which suggests an intense continental weathering event in the end of Nantuo glaciation[2]. Besides, the topmost of the Nantuo Formation also contain massive pyrite concretions which were pervasively distributed in the SCB.

All pyrite concretions for this work are collected in the top 0.5~10m of Nantuo formation. Most of the pyrite aggregation exists in the glacial diamictite and rare concretions are found inside the gravely siltstone/sandstone atop the glacial diamictite. In this study, pyrite concretions from four sections were analyzed, including one slope [Huakoushan (121.315°E, 32.024°N)] and three basin sections [Tongle (121.832°E, 29.539°N), Yazhai (121.874°E, 29.756°N) and Datan (121.763°E, 29.458°N)]. Pyrite concretions occur as elliptical nodules with their long axis parallel to the bedding surface. The diameter of pyrite concretions can be as large as 20 cm in basin sections, but is reduced to approximately 1 cm in shelf regions. Pyrite nodules are composed of densely packed euhedral pyrite crystals, no framboidal pyrite or framboidal cores are identified.

Supplementary Notes 2.Calculation for Mixing Model

The calculation for mixing model is based on mass balance models. [8, 9] Let c_1, c_2 and δ_1, δ_2 to be the concentration and δ value for two end members respectively. The elemental mass balance and isotopic mass balance can be written as:

$$\begin{cases} c = f \cdot c_1 + (1-f) \cdot c_2 \\ \delta \cdot c = f \cdot c_1 \cdot \delta_1 + (1-f) \cdot c_2 \cdot \delta_2 \end{cases} \tag{S1}$$

Here c and δ is the concentration and δ value for mixed system, *f* is the fraction for the first end member. Rearranging equation S1 we get:

$$\delta = -\frac{c_1 c_2 (\delta_1 - \delta_2)}{c_1 - c_2} \cdot \frac{1}{c} + \frac{c_1 \delta_1 - c_2 \delta_2}{c_1 - c_2} \tag{S2}$$

As a result, the δ and c in the mixing model are inversely proportional.

Supplementary Notes 3. Calculation for Rayleigh Distillation

The Rayleigh isotope distillation model is used to describe a gradual fractionation process for a closed system. [8, 10] It assumes a first order reaction while the rate factor for different isotope is unequal. Let A and B be different isotopes of an element, the rate equations for the reaction is written by:

$$\mathrm{d[A]} = -k_A\mathrm{[A]} \tag{S3}$$

$$\mathrm{d[B]} = -k_B\mathrm{[B]} \tag{S4}$$

Here the rate factor $k_A \neq k_B$, [A] and [B] represents the concentration for A and B respectively. Here we define the fractionation factor as the ratio of the rate constant:

$$\alpha = \frac{k_B}{k_A} \tag{S5}$$

Rearranging the equation, we get:

$$\frac{\mathrm{d[B]}}{\mathrm{[B]}} = \alpha \cdot \frac{\mathrm{d[A]}}{\mathrm{[A]}} \tag{S6}$$

Then integrating:

$$\frac{\mathrm{[B]}}{\mathrm{[B]_0}} = \left(\frac{\mathrm{[A]}}{\mathrm{[A]_0}}\right)^{\alpha} \tag{S7}$$

Here we can define the fraction of original mass remaining *f* as:

$$f = \frac{\mathrm{[A]}}{\mathrm{[A]_0}} \tag{S8}$$

So, f=1 means the initial state, f=0 means the termination. Divide both side by $\mathrm{[A]/[A]_0}$ then subtracting 1 in both sides, we get:

$$\frac{\mathrm{[B]/[A]} - \mathrm{[B]_0/[A]_0}}{\mathrm{[B]_0/[A]_0}} = f^{\alpha-1} - 1 \tag{S9}$$

Here we neglect the difference between $\mathrm{[B]_0/[A]_0}$ and $\mathrm{[B]_{STD}/[A]_{STD}}$, STD means isotopic reference material. Then the equation can be written as:

$$\frac{\mathrm{[B]/[A]} - \mathrm{[B]_{STD}/[A]_{STD}}}{\mathrm{[B]_{STD}/[A]_{STD}}} - \frac{\mathrm{[B]_0/[A]_0} - \mathrm{[B]_{STD}/[A]_{STD}}}{\mathrm{[B]_{STD}/[A]_{STD}}} = f^{\alpha-1} - 1 \tag{S10}$$

Replace B by ^{60}Ni, A by ^{58}Ni, then the formula could be written by:

$$\delta^{60}\mathrm{Ni_r} - \delta^{60}\mathrm{Ni_0} = 1000 \cdot (f^{\alpha-1} - 1) \tag{S11}$$

Here $\delta^{60}\mathrm{Ni_0}$ means the δ^{60}Ni value for initial material, $\delta^{60}\mathrm{Ni_r}$ means the δ^{60}Ni value for remaining Ni.

In a kinetic fractionation model for closed system, assuming the pyrite precipitate from seawater, the relationship between $\delta^{60}\mathrm{Ni_{py}}$, $\delta^{60}\mathrm{Ni_r}$, and $\delta^{60}\mathrm{Ni_{SW}}$ can be written as:

$$(1-f)\cdot\delta^{60}\mathrm{Ni_{py}}+f\cdot\delta^{60}\mathrm{Ni_{r}}=\delta^{60}\mathrm{Ni_{SW}} \quad \text{(S12)}$$

Here $\delta^{60}Ni_{py}$, $\delta^{60}Ni_{r}$, and $\delta^{60}Ni_{SW}$ stand for the average $\delta^{60}Ni$ value for pyrite, residual seawater and original seawater respectively. Replacing the $\delta^{60}Ni_{r}$, then we get:

$$(1-f)\cdot\delta^{60}\mathrm{Ni_{py}}+f\cdot\left(\delta^{60}\mathrm{Ni_{SW}}+1000\cdot\left(f^{\alpha-1}-1\right)\right)=\delta^{60}\mathrm{Ni_{SW}} \quad \text{(S13)}$$

Rearranging the equation, we get:

$$\delta^{60}\mathrm{Ni_{py}}=\delta^{60}\mathrm{Ni_{SW}}-1000\cdot\frac{f^{\alpha}-f}{1-f} \quad \text{(S14)}$$

Supplementary Notes 4. Method for Nickel Purification

In this work, the MQ H_2O used here were produced by Milli-Q Element system (Millipore, USA). The resistivity for MQ water is 18.6 MΩ. HCl, HNO_3 and HF used for experiment are all optima-grade, purchased from Beijing Institute of chemical Reagents (BICR). Acids are all distilled for at least once before used. The acetone used for the experiment was CMOS electronic grade, produced by Shanghai Hushi Laboratorial Equipment Co., Ltd. Dimethylglyoxime (DMG) was ACS reagent (⩾99%) produced by Sigma-Aldrich. All the instrument including beaker, tips and centrifuge tube was washed by HCl and HNO_3 before use.

The purification for Ni is conducted by 5 different column separation steps.[11] Since Fe, Ca, Mg, Ti would interfere nickel isotope measurement , it is necessary to isolate Ni before MC-ICP-MS measurement. Our method is developed from previous works. [2, 12, 13, 14] A summary for the Ni isolation procedure is shown in table S3.

(1) Removing Ca by concentrated HCl

1ml Bio-Rad AG50W-X8 cation exchange resin is used for this step. The resin was first washed by 2 x 5ml MQ H_2O, 2 x 5ml 6mol/L HCl then 2 x 5ml MQ H_2O, then conditioning with 1ml concentrated HCl twice. First, the sample was loaded onto the resin with 1ml solution in concentrated HCl. Ni starts eluting as soon as loaded to the resin, and it could be quantitatively collected using 6 x 1ml concentrated HCl to wash the resin. Residue elements in the resin could be completely washed off using 5 x 1ml 6mol/L HCl. The recovery rate for Ni is higher than 99.9%. Around 95% of Ca, 90% of Fe would be removed in this step.

(2) Removing Fe, Al, Ti by HF-HNO_3 mixed solution

The same column with step (1) was used for this step. The washing step is also same with step (1), but here 2 x 1ml 0.5mol/L HF + 1 mol/L HNO_3 mixed solution was used for conditioning. The sample was loaded to the resin using 0.5ml solution in 0.5mol/L HF + 1 mol/L HNO_3. Fe, Al, Ti would be washed off from the resin immediately as a complex with fluorinion. After completely washing Fe, Al, Ti using 5 x 0.5ml 0.5mol/L HF + 1 mol/L HNO_3 mixed solution, the residue elements including Ni was completely collected using 5 x 1ml 6mol/L HCl. The recovery rate for Ni is higher than 99.9%, while the removal of Fe, Al, Ti, Na is around 99%.

(3) Removing Fe, Mn by HCl-acetone solution

The column and the washing procedure for this step is same with that for step (1). 2 x 1ml 95%

acetone – 5% concentrated HCl was used for conditioning. The sample was first dissolved in 100μl concentrated HCl, then added 1.90ml acetone. If 100μl HCl cannot entirely dissolve the sample, more HCl was used, keeping the volume ratio for HCl:acetone to be 1:19. After loading the sample, 12 x 1ml 95% acetone – 5% concentrated HCl was used to wash Fe, Mn off the resin. Finally, 5 x 1ml 6mol/L HCl was used to collect the Ni. The recovery rate for Ni is higher than 99.9%, while almost all the Fe, Mn, Cu, Co, Cr would be removed after this step. This step is useful for removing large amount of Fe.

(4) Isolating Ni by dimethylglyoxime solution

DMG is a specific compound that chelate with Ni. $Ni(DMG)_2$ is a neutral complex which can't be adsorbed by cation exchange resin. The same column and washing procedure as step (1) are used for this step. Samples were loaded to the resin as a solution in 2ml 0.1mol/L DMG – 5% HCl – 95% acetone. Ni was immediately eluted from the resin. Then 5 x 1ml 0.1mol/L DMG – 5% HCl – 95% acetone solution was loaded for completely recovery of Ni. After Collecting Ni, at least 3 x 1ml acetone should be loaded to the column in order to wash off the remaining DMG. The recovery rate for Ni is around 98% for this step. Around 98% of Ca, Mg, 90% of Al, K, 10% of Ti would be removed in this step.

(5) Removing Fe by anion exchange resin

Since ^{58}Fe could interfere ^{58}Ni, the concentration for Fe must be critical low for the precision measurement of Ni isotope. So, for all the samples, a column separation for Fe using anion exchange resin is conducted in the final stage. 0.3ml Bio-Rad AG1-X8 anion exchange resin is used for this step. The resin was first washed by 5 x 1ml MQ H_2O, 5 x 1ml 6mol/L HCl then 5 x 1ml MQ H_2O, then conditioning with 4 x 0.5ml 6mol/L HCl. Then load the sample in 0.5ml 6mol/L HCl solution. Fe would be adsorbed in the resin as complex with chloridion, while Ni would be eluted immediately. After loading 3 x 0.5ml 6mol/L HCl, Ni would be completely washed out from the resin. The recovery rate for Ni is higher than 99.9% for this step.

In this work, samples were first digested using mixed solution of concentrated HCl and HNO_3. After measuring concentration using ICP-OES, sample solution containing 0.12μg to 0.5μg of Ni was taken out for Ni purification. Before separation, ^{61}Ni-^{62}Ni double spike were added to the sample. The $^{62}Ni_{spk}$:$^{58}Ni_{smp}$ for samples are 1.25 (First batch) or 1.50 (Second batch). The accuracy for the measurement has been confirmed for $^{62}Ni_{spk}$:$^{58}Ni_{smp}$ between 0.5 and 2.0. The column

separation process is (3)-(4)-(2)-(4)-(4)-(1)-(3)-(2)-(5). The total recovery rate for Ni is higher than 90%, while the concentration for all other elements are less than 1% of Ni.

In this work, nickel isotope for geological reference material including BHVO-2, BCR-2, NOD-A-1, NOD-P-1 were measured to monitor the accuracy. The $\delta^{60}Ni$ value for all reference material are agree with published data. (Tabel *S1*, Fig *S6*)

Supplementary Table S1. Nickel isotope data

Sample	Ni (ppm)	$\delta^{60}Ni$(‰)	2SD	Exp *
Huakoushan				
CYS-3	160.9	-0.13	—	2
CYS-banded	99.0	-0.21	—	2
HKS-2	160.2	-0.04	—	2
HKS-3	35.7	-0.74	—	1
HKS-4	113.0	-0.20	—	2
HKS-6 (1)	157.1	-0.40	—	1
HKS-6 (2)	160.2	-0.23	—	2
HKS-7	80.1	-0.30	—	1
HKS-8 (1)	41.1	1.52	—	1
HKS-8 (1)-rep [&]	41.1	1.39	—	2
HKS-8 (2)	33.0	0.84	—	2
Tongle				
TL-1-4	16.7	0.05	—	2
TL-1-5	17.6	0.05	—	2
TL-1-7	23.1	-0.05	—	2
TL-2	13.8	0.46	—	1
TL-4	23.0	0.00	—	1
Yazhai				
YZ-1-2	70.6	-0.43	—	2
YZ-2	101.5	-0.21	—	1
YZ-2-rep [&]	101.5	-0.40	—	2
YZ-2-1	71.1	-0.18	—	2
YZ-2-2	107.6	-0.01	—	2
YZ-2-3	91.9	-0.11	—	2
YZ-3	154.9	0.12	—	1
YZ-4	101.3	-0.18	—	1

Sample	Ni (ppm)	$\delta^{60}Ni$(‰)	2SD	Exp *
YZ-5-3	149.8	-0.11	—	2
Datan				
DT-1	15.0	0.47	—	1
DT-6	2.4	1.03	—	1
DT-9	22.0	0.42	—	1
Reference Material #				
	—	0.05		1
	—	0.07		1
BHVO-2	—	0.09	0.09	1
	—	-0.02		2
	—	0.07		2
BCR-2	—	0.18	—	2
	—	1.00		1
	—	1.02		1
NOD-A-1			0.09	
	—	0.96		1
	—	0.92		2
NOD-P-1	—	0.36	—	1

* Experiment batch.

& rep marks replicate samples.

Standard deviations for reference materials are employed to represent external precision of samples.

Supplementary Table S2. REE component and element concentration data (in ppm)

	Ni	Al	Ti	La	Ce	Pr	Nd	Pr	Sm	Eu	Gd	Dy	Y	Ho	Er	Tm	Yb	Lu
Huakoushan																		
CYS-2	15.30	4266.5	38.47	0.916	2.099	0.303	1.216	0.312	0.060	0.305	0.034	0.198	0.954	0.032	0.103	0.014	0.097	0.010
CYS-3	75.88	33455	225.7	4.983	14.67	2.019	7.991	2.238	0.352	2.090	0.232	1.248	6.313	0.238	0.640	0.080	0.537	0.068
CYS-b	72.51	13170	127.2	3.197	7.546	0.981	3.636	0.795	0.136	0.895	0.108	0.525	2.862	0.099	0.257	0.038	0.227	0.035
HKS-2	51.02	18117	154.9	2.983	8.795	1.362	6.338	1.622	0.261	1.310	0.116	0.495	2.449	0.079	0.252	0.033	0.211	0.023
HKS-3	17.98	9167.5	58.69	3.333	7.421	0.954	3.505	0.722	0.133	0.806	0.092	0.361	1.744	0.060	0.178	0.020	0.127	0.014
HKS-4	19.79	7286.8	98.03	1.102	2.595	0.353	1.488	0.511	0.115	0.645	0.089	0.469	2.899	0.094	0.273	0.035	0.184	0.025
HKS-6	75.28	30324	247.9	4.928	14.73	2.028	8.273	2.200	0.326	1.965	0.224	1.047	5.570	0.198	0.574	0.076	0.488	0.055
HKS-7	86.53	17028	157.3	3.808	9.292	1.279	4.985	1.113	0.195	1.034	0.131	0.655	3.331	0.117	0.335	0.045	0.255	0.038
HKS-8	24.44	7121.7	42.03	1.468	3.833	0.532	2.118	0.384	0.077	0.350	0.042	0.199	1.060	0.041	0.115	0.015	0.086	0.010
HKS-9	218.2	83609	580.5	12.01	34.22	4.927	21.94	5.915	0.913	5.098	0.585	2.773	13.18	0.498	1.398	0.148	1.088	0.151
Tongle																		
TL-1-1	7.489	12396	172.8	5.975	13.09	1.759	6.532	1.277	0.212	0.915	0.106	0.439	2.166	0.077	0.253	0.031	0.184	0.023
TL-1-2	8.053	10270	136.9	7.658	15.69	1.913	7.222	1.225	0.189	0.991	0.110	0.511	2.320	0.086	0.222	0.033	0.173	0.025
TL-1-3	9.362	11606	176.2	5.802	12.24	1.545	5.860	1.210	0.211	0.957	0.101	0.439	1.846	0.069	0.207	0.022	0.168	0.017
TL-1-4	11.95	11094	148.5	4.773	10.54	1.324	4.969	1.017	0.179	0.731	0.098	0.390	1.990	0.071	0.208	0.030	0.158	0.025
TL-1-5	13.25	12147	232.7	6.062	12.33	1.602	5.866	1.131	0.220	0.939	0.103	0.475	2.543	0.081	0.283	0.035	0.214	0.025
TL-1-6	13.03	14387	255.8	4.084	8.168	0.964	3.526	0.745	0.195	0.705	0.092	0.440	2.112	0.074	0.222	0.044	0.216	0.035
TL-1-7	18.46	12962	221.5	5.364	10.32	1.292	4.596	0.952	0.204	0.809	0.099	0.498	2.591	0.098	0.310	0.043	0.283	0.027
TL-2-1	16.90	6918.2	128.9	5.287	9.632	1.084	4.016	0.849	0.141	0.677	0.087	0.392	1.829	0.065	0.202	0.026	0.178	0.024
TL-2-2	12.97	7602.6	131.3	4.832	9.395	1.138	4.198	0.887	0.136	0.659	0.078	0.435	2.220	0.074	0.238	0.033	0.187	0.025
TL-2-3	13.76	11316	189.8	4.391	9.406	1.158	4.017	0.856	0.166	0.701	0.083	0.449	2.371	0.084	0.272	0.036	0.256	0.033
TL-2-4	12.19	13917	194.1	10.38	20.77	2.523	9.708	1.586	0.230	1.281	0.138	0.613	2.856	0.098	0.332	0.040	0.260	0.031
TL-2-5	18.44	8055.3	134.2	4.771	9.089	1.060	3.835	0.685	0.133	0.585	0.080	0.347	2.027	0.074	0.205	0.029	0.194	0.020
TL-4-1	12.79	12331	177.8	4.648	8.970	1.077	3.871	0.657	0.138	0.622	0.075	0.397	1.937	0.070	0.237	0.026	0.184	0.017
TL-4-2	11.38	11395	167.8	4.434	8.223	1.003	3.832	0.723	0.127	0.613	0.067	0.335	1.846	0.061	0.185	0.028	0.167	0.017
TL-4-3	11.06	9905.4	190.3	3.014	5.869	0.721	2.528	0.496	0.110	0.549	0.069	0.399	2.441	0.088	0.260	0.038	0.267	0.032

	Ni	Al	Ti	La	Ce	Pr	Nd	Pr	Sm	Eu	Gd	Dy	Y	Ho	Er	Tm	Yb	Lu
Yazhai																		
YZ-1-1	25.02	14833	278.3	7.477	14.61	1.713	5.731	1.055	0.185	0.979	0.097	0.359	1.513	0.050	0.176	0.018	0.122	0.016
YZ-1-2	43.54	18090	367.6	8.335	16.05	1.895	6.606	1.212	0.227	1.257	0.112	0.487	2.009	0.070	0.231	0.036	0.195	0.021
YZ-1-3	30.71	16522	333.3	12.82	25.33	3.084	10.53	1.878	0.348	1.916	0.177	0.716	3.243	0.117	0.369	0.049	0.288	0.041
YZ-2-1	50.28	14496	359.1	8.829	18.08	2.165	8.416	1.400	0.276	1.384	0.151	0.643	2.995	0.111	0.317	0.035	0.261	0.034
YZ-2-2	70.95	12309	275.8	6.973	14.30	1.741	5.846	1.230	0.233	1.274	0.152	0.616	3.047	0.110	0.308	0.039	0.260	0.028
YZ-2-3	59.26	16683	379.5	9.054	18.72	2.208	7.839	1.531	0.296	1.565	0.185	0.725	3.675	0.119	0.323	0.047	0.302	0.041
YZ-4-1	31.77	17339	371.8	8.048	16.36	1.954	7.044	1.422	0.297	1.378	0.136	0.638	2.891	0.117	0.302	0.047	0.241	0.035
YZ-4-2	42.53	17291	360.4	7.358	13.95	1.748	6.624	1.222	0.258	1.111	0.122	0.514	2.507	0.092	0.287	0.032	0.209	0.026
YZ-4-3	72.08	11201	228.0	7.992	15.74	1.811	6.501	1.210	0.229	1.249	0.127	0.527	2.301	0.076	0.247	0.031	0.190	0.024
YZ-5-1	85.95	10971	270.4	3.884	8.723	1.143	4.489	0.963	0.184	0.934	0.101	0.462	2.317	0.079	0.236	0.030	0.204	0.021
YZ-5-2	78.38	6744.2	167.6	3.052	6.659	0.811	3.143	0.569	0.115	0.570	0.058	0.282	1.498	0.054	0.146	0.021	0.126	0.014
YZ-5-3	119.1	6240.5	142.4	2.186	4.822	0.618	2.474	0.483	0.075	0.431	0.052	0.240	1.284	0.043	0.139	0.018	0.116	0.010
Datan																		
DT-2	5.196	8135.5	113.9	7.134	16.58	2.302	8.843	2.055	0.360	1.739	0.170	0.614	2.476	0.090	0.266	0.033	0.200	0.028
DT-3	22.09	10072	147.8	19.46	39.69	4.970	19.26	3.773	0.578	3.081	0.243	0.847	2.769	0.111	0.341	0.028	0.193	0.023
DT-4	18.20	7492.4	119.2	40.37	84.70	10.42	39.88	7.735	1.322	6.122	0.499	1.368	3.470	0.157	0.473	0.033	0.244	0.022
DT-5	20.60	8888.4	131.6	13.06	27.08	3.455	13.25	2.554	0.482	2.076	0.183	0.629	2.106	0.084	0.248	0.025	0.154	0.018
DT-6	31.15	9209.8	130.1	14.59	30.80	3.863	15.22	3.403	0.567	2.796	0.252	0.910	3.561	0.127	0.386	0.044	0.265	0.033
DT-7	15.04	5923.4	103.9	8.599	18.77	2.424	10.46	2.323	0.382	1.734	0.143	0.552	2.044	0.079	0.223	0.025	0.155	0.018
DT-8	37.12	11092	150.7	18.70	38.43	5.043	19.32	3.422	0.553	3.136	0.295	1.072	4.493	0.184	0.545	0.059	0.406	0.054
DT-9	27.25	3465.4	40.87	4.051	9.043	1.200	4.606	0.864	0.133	0.735	0.063	0.228	0.891	0.037	0.100	0.011	0.097	0.009
DT-10	11.89	5712.7	64.81	6.369	15.30	1.943	7.528	1.627	0.278	1.501	0.148	0.627	2.450	0.083	0.238	0.020	0.160	0.019
DT-11	10.97	15852	149.7	9.006	20.49	2.701	10.68	2.201	0.357	2.388	0.227	0.798	2.461	0.105	0.300	0.032	0.211	0.031
DT-15	6.205	4848.0	42.61	13.63	27.52	3.523	14.01	2.256	0.337	2.687	0.220	0.672	2.162	0.077	0.275	0.019	0.155	0.019
DT-17	10.42	2357.0	27.16	11.10	23.87	2.946	11.57	2.222	0.415	2.648	0.238	0.907	3.155	0.120	0.369	0.044	0.279	0.040

Supplementary Table S3. Ni purification procedure

C1 (1ml AG50W-X8)	Removing Ca Using Concentrated HCl	Volume
Conditioning	Concentrated HCl	2 x 1ml
Loading & Collecting	Concentrated HCl	1ml
Collecting	Concentrated HCl	6 x 1ml
C2 (1ml AG50W-X8)	Removing Ti, Al, Fe Using HF-HNO_3	
Conditioning	0.5mol/L HF + 1mol/L HNO_3	2 x 1ml
Loading Sample	0.5mol/L HF + 1mol/L HNO_3	0.5ml
Washing	0.5mol/L HF + 1mol/L HNO_3	5 x 0.5ml
Collecting	6mol/L HCl	5 x 1ml
C3 (1ml AG50W-X8)	Removing Fe Using HCl - Acetone	
Conditioning	95% Acetone + 5% Concentrated HCl	2 x 1ml
Loading Sample	95% Acetone + 5% Concentrated HCl	2ml
Washing	95% Acetone + 5% Concentrated HCl	12 x 1ml
Collecting	6mol/L HCl	5 x 1ml
C4 (1ml AG50W-X8)	Separating Ni Using DMG - HCl - Acetone	
Conditioning	0.1mol/L DMG + 95% Acetone + 5% HCl	2 x 1ml
Loading & Collecting	0.1mol/L DMG + 95% Acetone + 5% HCl	1ml
Collecting	0.1mol/L DMG + 95% Acetone + 5% HCl	6 x 1ml
Cleaning Resin	Acetone	3 x 1ml
C5 (0.3ml AG1-X8)	Removing Fe Using Anion Exchange Resin	
Conditioning	6mol/L HCl	4 x 0.5ml
Loading & Collecting	6mol/L HCl	0.5ml
Collecting	6mol/L HCl	3 x 0.5ml

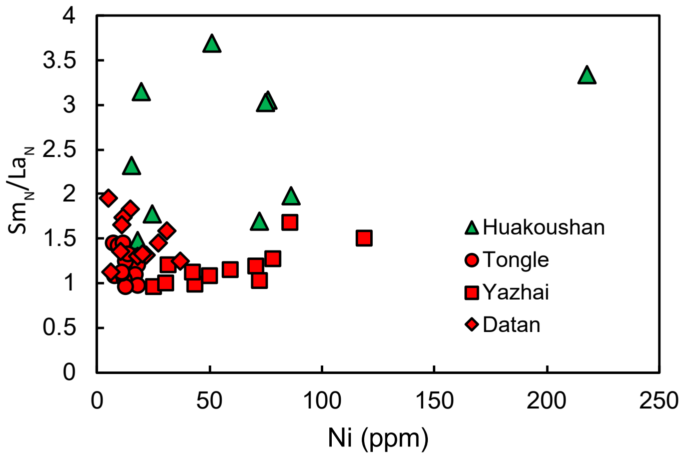

Supplementary Figure S1. Cross-plot of Ni concentration versus Sm_N/La_N for Nantuo pyrite samples. Here Sm and La are used to represent MREE and LREE respectively. The concentration for Sm and La are normalized by post-Archean Australian shale (PAAS). Here, green triangle, red circles, red square and red diamond represent Huakoushan, Tongle, Yazhai and Datan section respectively. The Huakoushan section is in slope region, while the other three section are in basin region. No relationship occurs between Ni concentration and Sb_N/La_N.

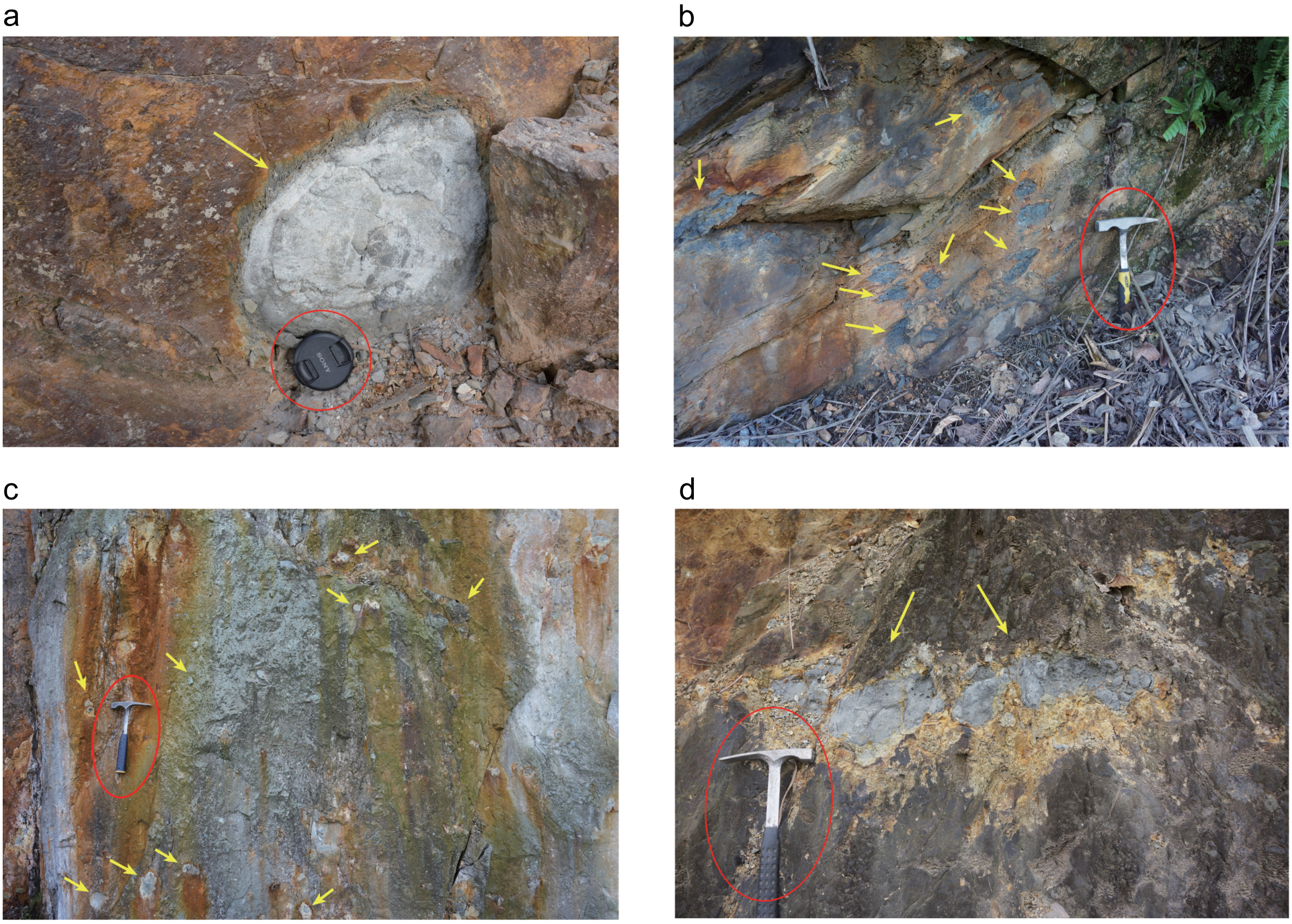

Supplementary Figure S2. Outcrop photographs for pyrite concretions. The pyrite concretion is pointed by yellow arrows. (a) Tongle Section; (b) Yazhai Section; (c) and (d) Huakoushan Section.

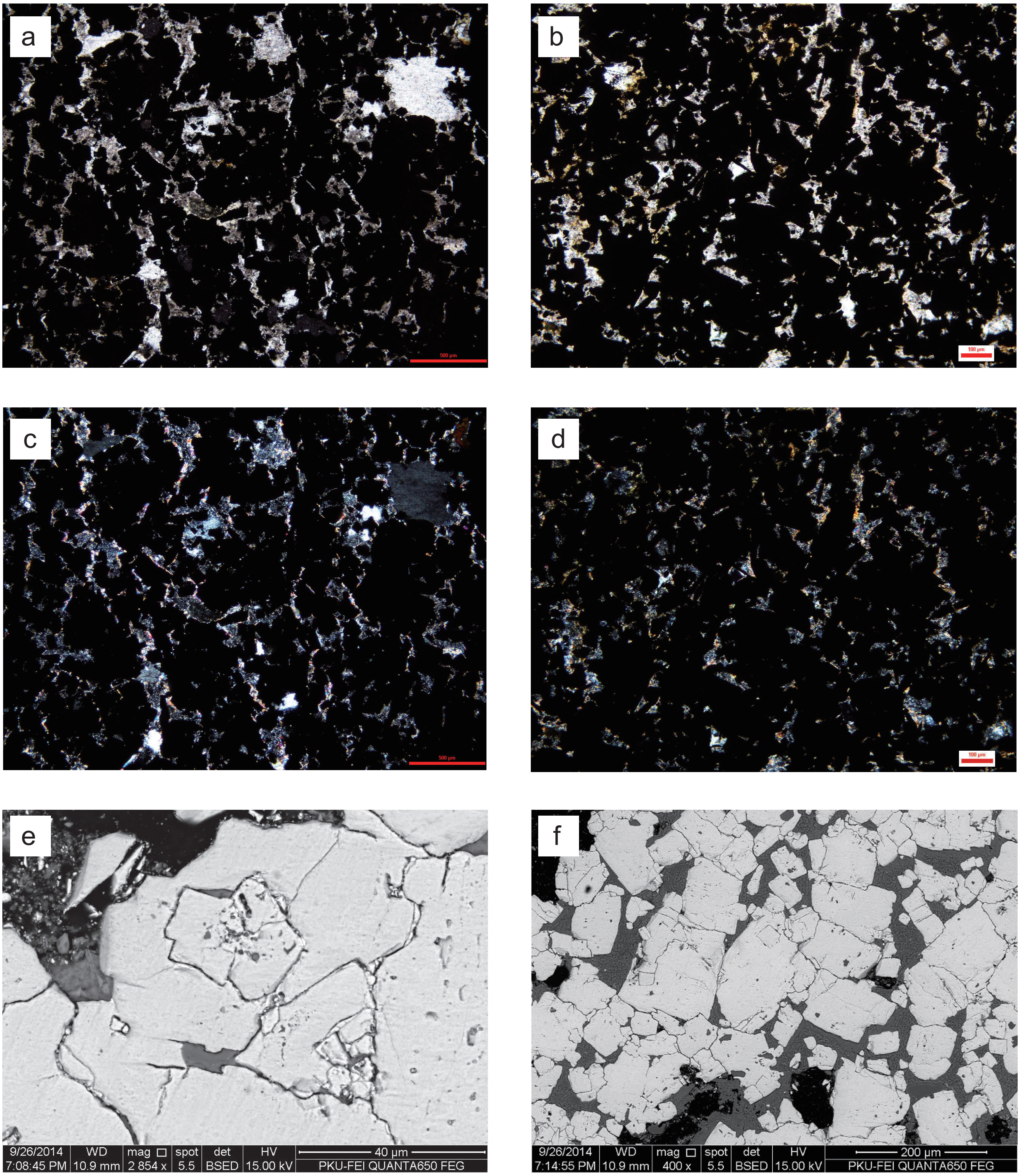

Supplementary Figure S3. Micrographs for Nantuo pyrite. (a) and (b) Under monopolar light, pyrite are opaque crystals, which is cemented by silicate materials; (c) and (d) Under orthogonal light, silicate minerals and silica-clastic (feldspar) could be better recognized. (a) and (c) is sample from Tongle section while (b) and (d) is sample from Yazhai section. (e) and (f) Electron back scattered diffraction (BSD) figure for sample from Tongle section. No framboidal pyrite or framboidal core is found in Nantuo pyrite.

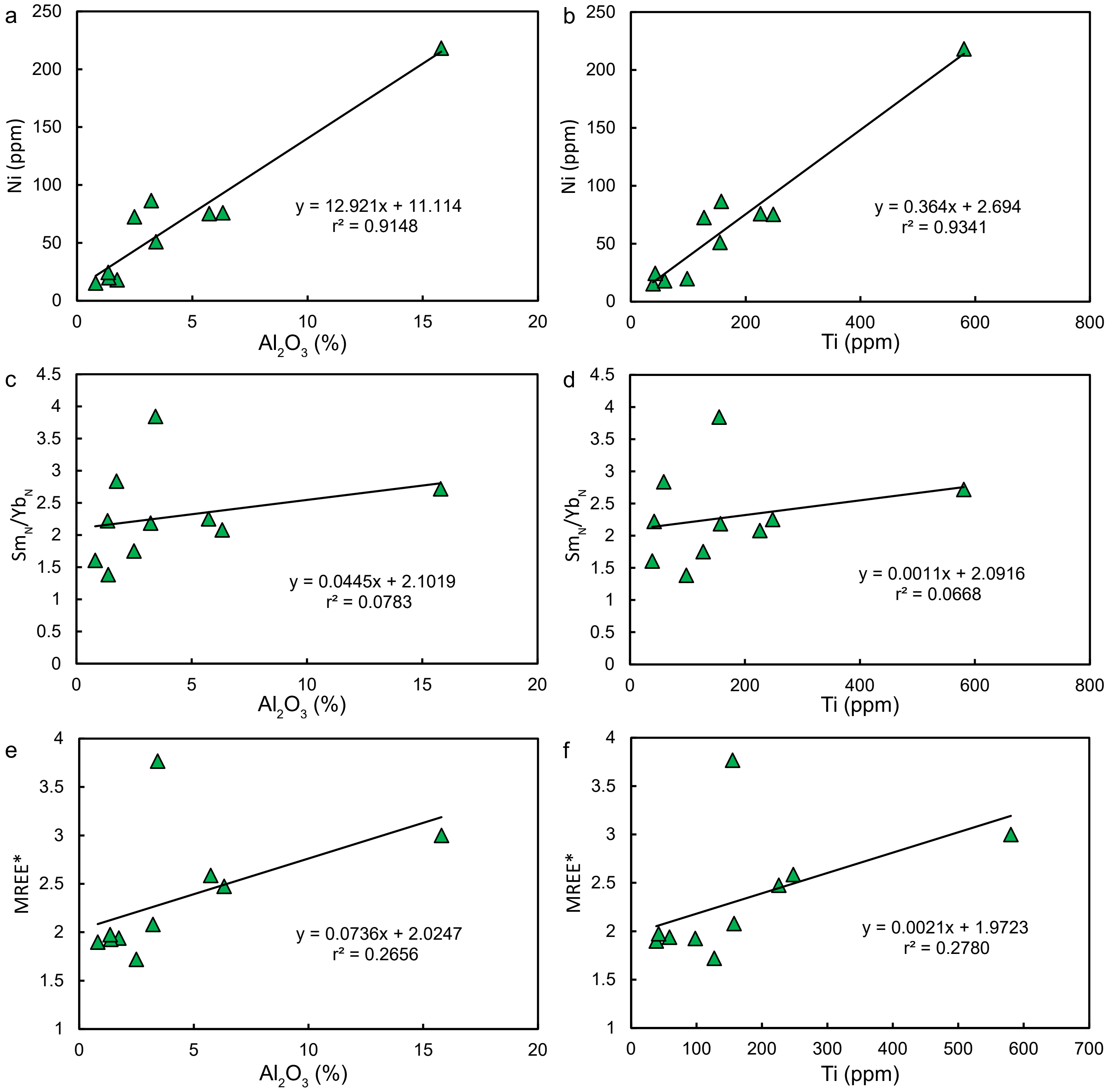

Supplementary Figure S4. Cross-plot of Ni concentration, Sm_N/Yb_N and MREE* versus Al_2O_3 and Ti contents for slope samples. (a) Cross-plot of Ni concentration versus Al_2O_3 contents for slope samples. (b) Cross-plot of Ni concentration versus Ti concentration for slope samples. (c) Cross-plot for Sm_N/Yb_N versus Al_2O_3 contents for slope samples. (d) Cross-plot for Sm_N/Yb_N versus Ti concentration for slope samples. (e) Cross-plot for MREE* versus Al_2O_3 contents for slope samples. (f) Cross-plot for MREE* versus Ti concentration for slope samples. Here La, Sm and Yb are used to represent LREE, MREE, HREE respectively. The MREE* is defined as $(2*Sm_N)/(La_N+Yb_N)$. All REE data are normalized by shale. Strong positive relationship occurs between Ni, MREE* and Al, Ti, suggesting the Ni and REE in slope samples are mainly from clay.

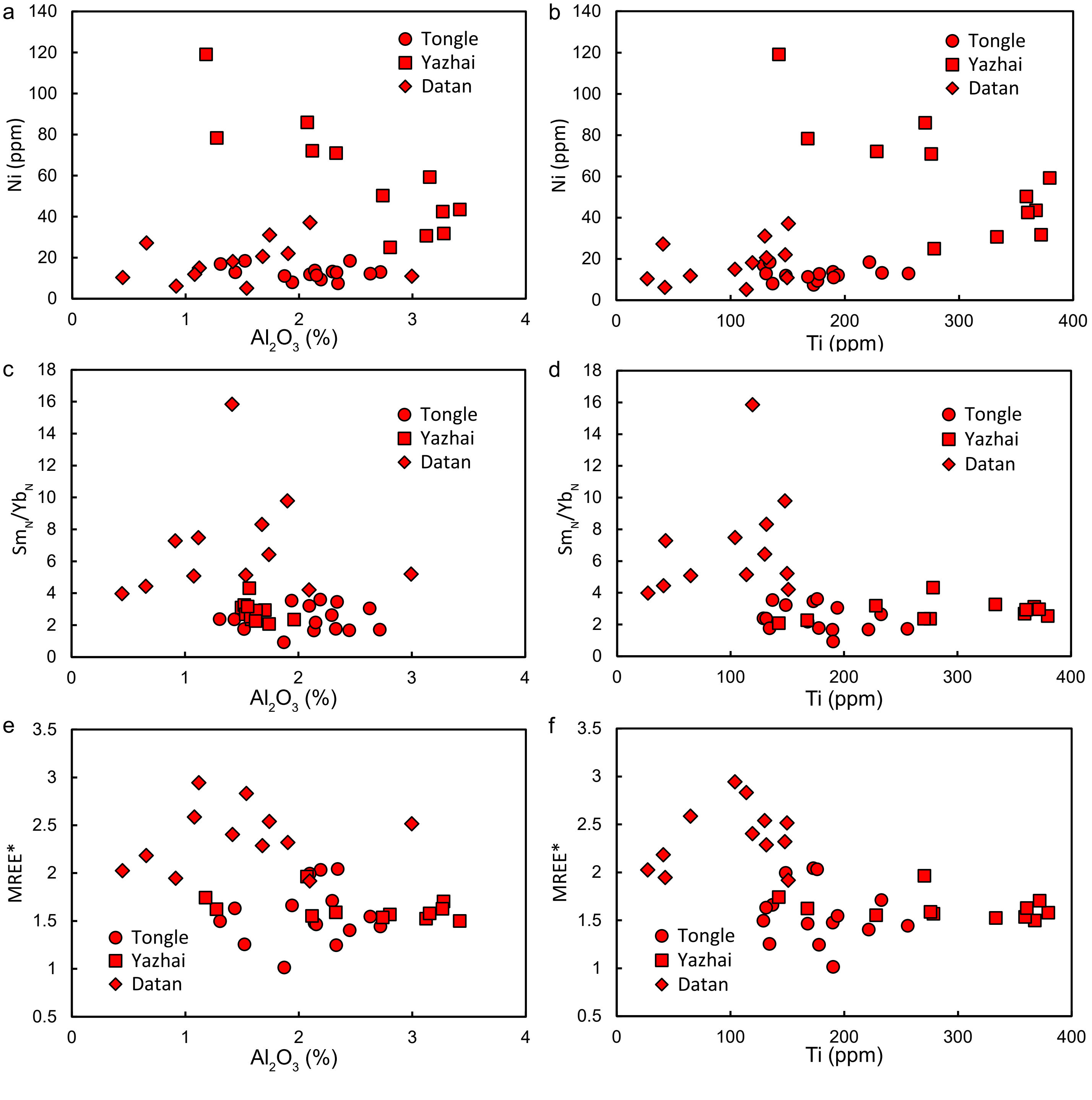

Supplementary Figure S5. Cross-plot of Ni concentration, Sm_N/Yb_N and MREE* versus Al_2O_3 and Ti contents for basin samples. (a) Cross-plot of Ni concentration versus Al_2O_3 contents for basin samples. (b) Cross-plot of Ni concentration versus Ti concentration for basin samples. (c) Cross-plot for Sm_N/Yb_N versus Al_2O_3 contents for basin samples. (d) Cross-plot for Sm_N/Yb_N versus Ti concentration for basin samples. (e) Cross-plot for MREE* versus Al_2O_3 contents for basin samples. (f) Cross-plot for MREE* versus Ti concentration for basin samples. Here La, Sm and Yb are used to represent LREE, MREE, HREE respectively. The MREE* is defined as $(2*Sm_N)/(La_N+Yb_N)$. The red circle, red square and red diamond correspond to Tongle, Yazhai and Datan section respectively. All REE data are normalized by shale. No relationship occurs in all relationship above, suggesting the contamination for Ni and REE for basin samples is weak.

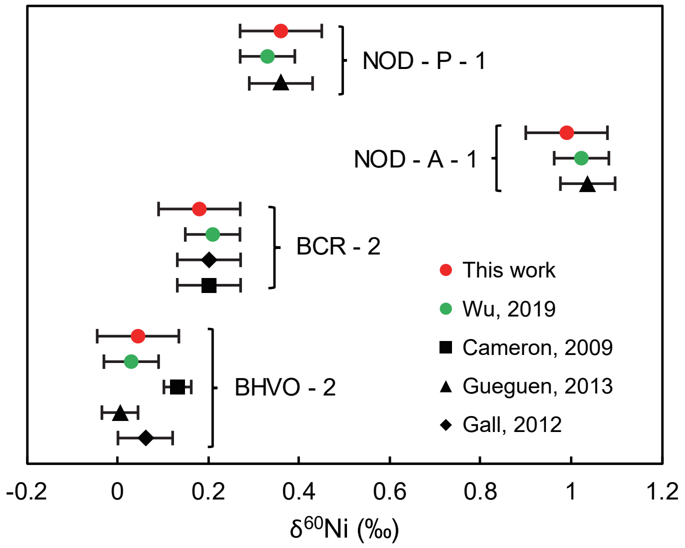

Supplementary Figure S6. δ^{60}Ni value for geological reference material. The red circle represents data in this work. The green circle represents published data from the same laboratory [11]. The black square, black triangle and black diamond are literature value [12, 14, 15]. The error bar is defined as 2SD (two standard deviation). The δ^{60}Ni for geological reference material in our work are close to literature value, which certify the accuracy for nickel isotopic measurement.

Supplementary Reference

1. Li ZX, Bogdanova SV, Collins AS, Davidson A, De Waele B, Ernst RE*, et al.* Assembly, configuration, and break-up history of Rodinia: A synthesis. *Precambrian Research* 2008, **160**(1-2)**:** 179-210.
2. Huang K-J, Teng F-Z, Shen B, Xiao S, Lang X, Ma H-R*, et al.* Episode of intense chemical weathering during the termination of the 635 Ma Marinoan glaciation. *Proceedings of the National Academy of Sciences of the United States of America* 2016, **113**(52)**:** 14904-14909.
3. Zhang Q-R, Chu X-L, Feng L-J. Chapter 32 Neoproterozoic glacial records in the Yangtze Region, China. *Geological Society, London, Memoirs* 2011, **36**(1)**:** 357-366.
4. Lang X, Chen J, Cui H, Man L, Huang K-J, Fu Y*, et al.* Cyclic cold climate during the Nantuo Glaciation: Evidence from the Cryogenian Nantuo Formation in the Yangtze Block, South China. *Precambrian Research* 2018, **310:** 243-255.
5. Lang X, Shen B, Peng Y, Xiao S, Zhou C, Bao H*, et al.* Transient marine euxinia at the end of the terminal Cryogenian glaciation. *Nature communications* 2018, **9**(1)**:** 3019.
6. Condon D, Zhu M, Bowring S, Wang W, Yang A, Jin Y. U-Pb Ages from the Neoproterozoic Doushantuo Formation, China. *Scinece* 2005, **308:** 95-98.
7. Zhang S, Jiang G, Han Y. The age of the Nantuo Formation and Nantuo glaciation in South China. *Terra Nova* 2008, **20**(4)**:** 289-294.
8. Li F-B, Teng F-Z, Chen J-T, Huang K-J, Wang S-J, Lang X-G*, et al.* Constraining ribbon rock dolomitization by Mg isotopes: Implications for the 'dolomite problem'. *Chemical Geology* 2016, **445:** 208-220.
9. Peng Y, Shen B, Lang X-G, Huang K-J, Chen J-T, Yan Z*, et al.* Constraining dolomitization by Mg isotopes: A case study from partially dolomitized limestones of the middle Cambrian Xuzhuang Formation, North China. *Geochemistry, Geophysics, Geosystems* 2016, **17**(3)**:** 1109-1129.
10. White WM. *Geochemistry*. Wiley-Blackwell, 2013.
11. Wu G, Zhu J-M, Wang X, Han G, Tan D, Wang S-J. A novel purification method for high precision measurement of Ni isotopes by double spike MC-ICP-MS. *Journal of Analytical Atomic Spectrometry* 2019, **34**(8)**:** 1639-1651.
12. Gall L, Williams H, Siebert C, Halliday A. Determination of mass-dependent variations in nickel isotope compositions using double spiking and MC-ICPMS. *J Anal At Spectrom* 2012, **27**(1)**:** 137-145.
13. Chernonozhkin SM, Goderis S, Lobo L, Claeys P, Vanhaecke F. Development of an isolation procedure and MC-ICP-MS measurement protocol for the study of stable isotope ratio variations of nickel. *J Anal At Spectrom* 2015, **30**(7)**:** 1518-1530.
14. Gueguen B, Rouxel O, Ponzevera E, Bekker A, Fouquet Y. Nickel Isotope Variations in Terrestrial Silicate Rocks and Geological Reference Materials Measured by MC-ICP-MS. *Geostandards and Geoanalytical Research* 2013, **37**(3)**:** 297-317.
15. Cameron V, Vance D, Archer C, House CH. A biomarker based on the stable isotopes of nickel. *Proceedings of the National Academy of Sciences of the United States of America* 2009, **106**(27)**:** 10944-10948.
